# Optimized control, and experimental validation of a novel multi-stage parabolic trough collector for solar water heating systems

**DOI:** 10.1038/s41598-025-34564-5

**Published:** 2026-01-22

**Authors:** Roba Tarek AbdelFatah, Raafat Shalaby, Irene Samy Fahim, Mohamed Mahran Kasem

**Affiliations:** 1 Smart Engineering Systems Research Center (SESC), School of Engineering and Applied Sciences, Nile University, Shaikh Zayed City, 12588 Egypt; 2 Aerospace Engineering Department, Cairo University, Giza, 12613 Egypt

**Keywords:** Solar water heaters, Multi-stage collectors (MPTC), Optimal control, Solar energy systems, Energy science and technology, Engineering

## Abstract

This study presents the development, optimized PID control, and experimental validation of a novel Multi-Stage Parabolic Trough Collector (MPTC) for solar water heating systems, aiming to enhance thermal efficiency and adaptability under varying environmental conditions. The research is structured into three key stages. First, a time-dependent mathematical model is developed to characterize the thermal and dynamic behavior of the system, incorporating essential parameters such as heat absorption, fluid dynamics, and energy losses. Second, a proportional-integral-derivative (PID) controller is designed and optimized using advanced techniques, including Genetic Algorithm (GA) and Particle Swarm Optimization (PSO) to ensure robust system stability and dynamic responsiveness. GA and PSO are selected over classical methods (e.g., Ziegler-Nichols) due to their superior ability to handle non-linearities, multi-objective trade-offs, and real-world disturbances typical of solar thermal systems. Lastly, a prototype MPTC featuring a modular, high-efficiency absorber design, is fabricated and experimentally tested under both controlled and real-outdoor conditions. Experimental results demonstrated a steady-state temperature error of 1.111% under dynamic irradiance conditions, confirming the model’s accuracy. These results are compared with simulation outputs to validate the model and fine-tune the control strategies. The present model achieves a thermal efficiency of 16.2%, which exceeds that of comparable models reported in the literature. This integrated approach bridges theoretical modeling with experimental validation, offering a practical and scalable framework for enhancing the performance of adaptive solar thermal systems.

**Supplementary Information:**

The online version contains supplementary material available at 10.1038/s41598-025-34564-5.

## Introduction

### Background and motivation

The global shift toward renewable energy has intensified research into solar water heating systems (SWHs), due to their strong potential for both residential and industrial applications^1^. However, these systems face significant challenges, including fluctuating solar irradiance and inefficient thermal storage, which undermine their reliability and overall performance^2^.

The design and control of SWHs have evolved significantly in response to the growing demand for renewable energy solutions. These systems utilize thermal energy to produce hot water, providing an environmentally friendly alternative to conventional heating methods. The literature on solar thermal systems highlights the importance of optimizing key components, such as collector designs^3^, heat transfer mechanisms^4^, storage tanks^1^, and control systems^5^, to enhance efficiency and adaptability to variable solar availability. Many Studies focus on mathematical modeling^6,7^ and computational techniques, such as Computational Fluid Dynamics (CFD) to enhance thermal storage performance, energy transfer, and fluid flow dynamics within these systems^8^.

### System configurations

In recent years, different water cycle configurations have been developed to optimize the performance of SWHs^2^.

**Fig. 1:**
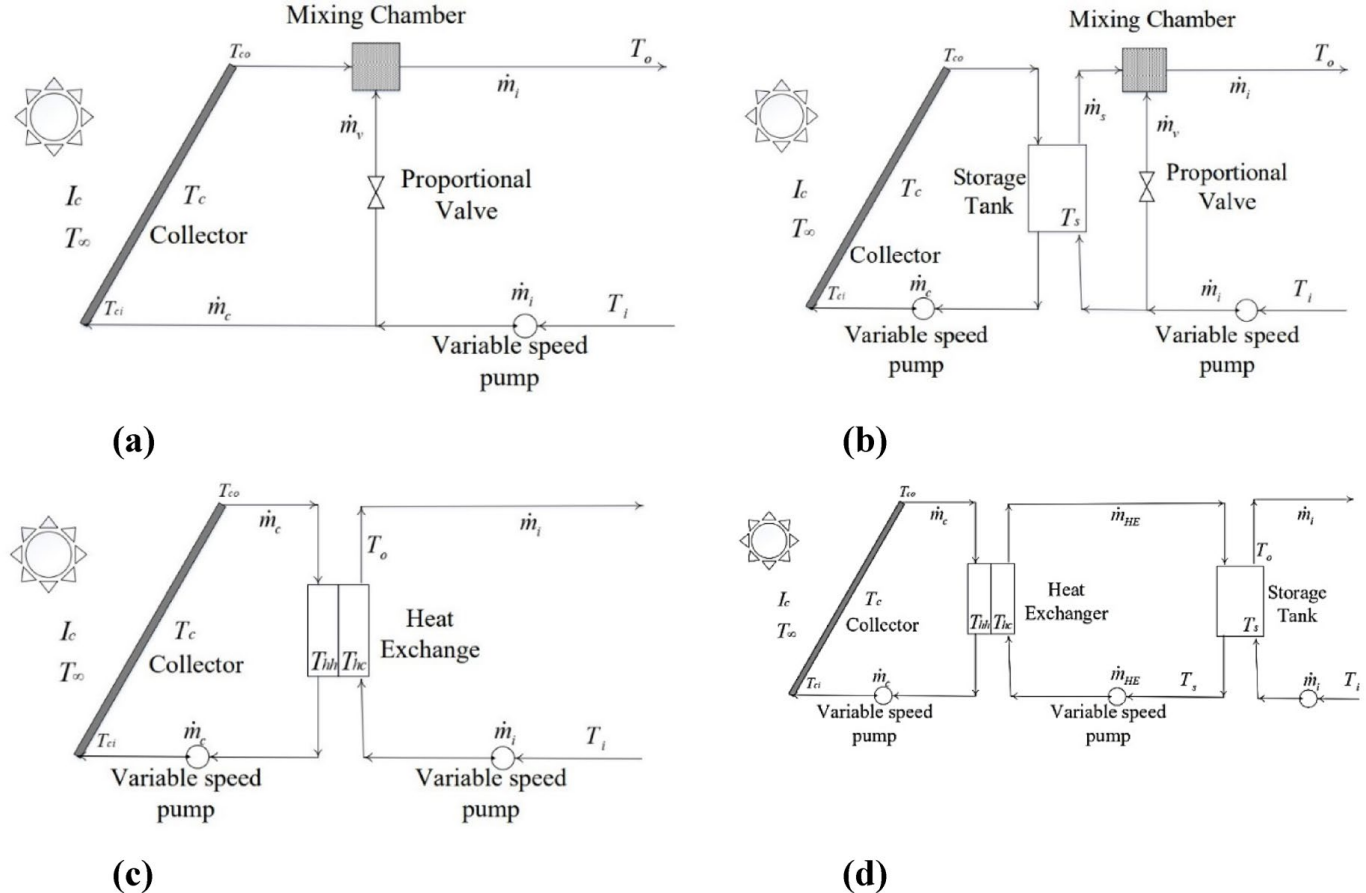
The different configurations of the SWHS^2^: (**A**) direct water feed to SWH, (**B**) Tank water feed to SWH, (**C**) Heat exchanger between SWH and the water in the storage tank, and (**D**) Heat exchanger between SWH and the water to be fed to the storage tank.

Figure 1 compares four common SWH configurations, each presenting distinct trade-offs^9,10^:

- **Direct feed** (Fig. 1a): Simple design, but susceptible to temperature fluctuations.
- **Tank-buffered** (Fig. 1b): Provides stable output temperature but incurs higher thermal losses.
- **Heat exchanger-based** (Fig. 1c/d): Enables efficient energy transfer, though it requires more complex control strategies.

The system illustrated in Fig. 1a uses hot water directly from the collector for the user, with cold water from the main supply stream added and mixed in a mixing chamber to achieve the desired temperature^9^. One pump circulates water between the solar collector and the storage tank, while another pump delivers cold input water to the storage tank. This configuration is shown in Fig. 1b. The storage tank acts as a high-capacity thermal buffer, ensuring a consistent water temperature even during fluctuations in solar radiation caused by erratic weather. Based on the energy balance principle, the tank maintains a steady energy supply by storing surplus thermal energy when not in use and reintroducing it into the system as needed^2^.

A closed-loop system is shown in Fig. 1c, where one pump circulates water between the heat exchanger and the solar collector, and another pump transports cold intake water from the thermal load to the heat exchanger. Similar to the storage tank in the previous system, the heat exchanger serves as a high-capacity thermal buffer, transferring thermal energy from the solar collector loop to the thermal load loop^9^.

Finally, Fig. 1d depicts a design where thermal energy is transferred from the solar collector to an intermediate loop via the heat exchanger, and subsequently to the thermal load through the storage tank. The combined thermal capacities of the heat exchanger and storage tank ensure stable thermal performance. This arrangement facilitates efficient energy transfer through conduction and convection in the heat exchanger, while the stabilizing tank provides thermal storage^10^.

### Literature review

Control strategies for SWHs have emerged as a critical area of research aimed at enhancing energy efficiency, system reliability, and user comfort. The literature highlights the use of various dynamic control techniques, including PI^6,7^, PID^11^, optimal control^12^, predictive control^13^ and feedforward control^14^ to regulate the temperature of the heat transfer fluid (HTF) and enhance overall system performance^14^.

Recent advancements in SWH controls strategies can be broadly categorized into three main areas:

1. **Algorithmic optimization (e.g., GA/PSO**^15^, **fuzzy logic**^16^**)** → offers improved system stability but often lacks real-time adaptability.
2. **Hardware innovations (e.g., FPGA**^17^, **microcontrollers**^18^**)** → provide high precision but can be costly and less scalable.
3. **Hybrid approaches (e.g., AI-driven**^19^**)** → show great promise but still require experimental validation under dynamic environmental conditions.

Modern control systems increase thermal performance and adapt to changing environmental and operational conditions. Historical control mechanism viewpoints, as articulated by Norton and Norton^20^, provided fundamental knowledge on the operational capabilities and limitations of early SWH systems. These studies paved the way for further developments, such as the microcontroller-based control systems developed by Huang^18^ and Jiang-tao^21^, which significantly improved user control and system automation.

Incorporation of optimization algorithms into control frameworks has recently attracted much attention, especially in the context of important works by Dennis^22^ and Dgany et al.^23^. Their studies demonstrated that both open-loop and closed-loop flow control systems can be effectively applied to regulate thermal storage and to manage energy distribution. Moreover, Lu’s^15^ comparative analysis of genetic algorithms (GA) and particle swarm optimization (PSO) highlighted the potential to achieve an optimal balance between responsiveness and stability in system performance.

Innovative technologies, such as step motor-driven mixing control systems explored by Cui^24^ and FPGA-based systems analyzed by Duan^17^, highlighted the critical role of advanced hardware in enhancing control accuracy. These technologies demonstrated the capability to adapt swiftly to dynamic environmental conditions, ensuring optimal thermal efficiency. Finally, research on application-specific topics, such as condensation control in flat-plate SWHs by Oshikiri and Anderson^25^, and the retrofitting of conventional heaters to solar systems by Bernardo and Bernardo^26^, demonstrates the adaptability of modern control techniques to diverse scenarios. These advancements underscore the importance of developing tailored control solutions to effectively address diverse user requirements and environmental conditions.

Bharathi et al.^19^ introduced a hybrid solar water heating model based on deep learning, enabling real-time optimization of energy extraction in response to fluctuating solar intensities. This advanced control approach demonstrated the potential of artificial intelligence to improve system performance. Similarly, Mao et al.^27^ emphasized the importance of advanced control strategies in eco-friendly buildings, utilizing predictive algorithms to reduce energy loss and improve thermal management.

Alaskaree and Breesam^28^ emphasized the significant role of electronic control systems in enhancing the efficiency of SWHs. Their study demonstrated that advanced controllers, such as PID and fuzzy logic systems, effectively regulate heat and reduce energy losses. Similarly, Ahmed et al.^16^ applied fuzzy logic control to optimize photovoltaic-thermal solar water collectors, resulting in improved thermal performance and increased energy conversion efficiency. Collectively, these studies highlight the pivotal role of advanced control methodologies in transforming solar water heating systems. Through the integration of intelligent algorithms, technological innovations, and optimization techniques, researchers continue to advance the efficiency, sustainability, and reliability of solar thermal applications.

### Motivation and innovation

Zhao et al.^29^ developed a novel cascaded PTC featuring multiple concentration ratios to enhance overall optical efficiency compared to conventional designs. Similarly, the present study introduces a novel multi-stage PTC aimed at improving solar water heating performance, particularly for residential applications. The key distinction between the two models lies in their intended use: Zhao et al.'s design targets large-scale power generation, whereas the proposed system in this work is specifically optimized for small-scale, household solar heating applications.

The present study introduces a **novel modular Multi-Stage Parabolic Trough Collector (MPTC)** designed to enhance thermal performance and adaptability for residential solar water heating applications. Unlike conventional single-stage PTCs, the proposed system features a **multi-stage configuration**, where multiple parabolic segments are aligned in series along a shared aperture. This design enables **incremental thermal gain** and **improved absorber tube utilization** without expanding the system’s footprint. A comprehensive, **time-dependent thermal model** is developed, incorporating nodal discretization of the absorber tube and real environmental variables (e.g., solar irradiance, ambient temperature). Together, optical and thermal innovations, in this design, offer a scalable and adaptable solution for improving solar energy capture and storage.

In addition to system design, the study contributes a **hybrid control optimization framework** that employs both **Genetic Algorithm (GA)** and **Particle Swarm Optimization (PSO)** to fine-tune PID controllers for two MPTC configurations—with and without thermal storage tanks. A custom multi-objective cost function is introduced, balancing steady-state error, overshoot, settling time, and dynamic penalties. Comparative results demonstrate distinct advantages of GA (greater precision) and PSO (faster response), guiding application-specific controller selection. The simulation model is experimentally validated using a fabricated MPTC prototype tested under dynamic outdoor conditions. Results highlight strong agreement between simulation and measured data, while also revealing the impact of real-world disturbances (e.g., wind cooling). The study is further distinguished by a **quantitative comparison with existing literature**, confirming improved control response and energy gains over prior systems. Collectively, these contributions establish a practical and innovative framework for optimizing solar thermal performance through integrated design, control, and validation.

The proposed MPTC design achieves **16.2% thermal efficiency** and **28.5% higher outlet temperature** compared to conventional single-stage PTCs. Unlike existing systems, it integrates a multi-stage optical configuration with metaheuristic-tuned PID control, enabling improved adaptability to fluctuating irradiance and ambient conditions.

The paper is structured as follows: First, a comprehensive literature review is conducted to establish the foundation for developing a time-dependent mathematical model that captures the thermal and dynamic behavior of the solar water heating system. The model integrates key parameters such as heat absorption, fluid dynamics, and energy losses, providing a robust framework for system analysis.

Next, a proportional-integral-derivative (PID) controller is designed and optimized using advanced techniques, including GA and PSO. These algorithms are employed to determine the optimal control parameters, ensuring system stability and high performance under varying environmental conditions.

Finally, the study advances to the experimental phase, where a prototype of the proposed MPTC is fabricated. The system’s performance is evaluated under both controlled laboratory settings and real-world conditions to validate the mathematical model and the effectiveness of the control strategies. Experimental results are compared with simulation outputs to assess accuracy and identify areas for further optimization.

**Fig. 2:**
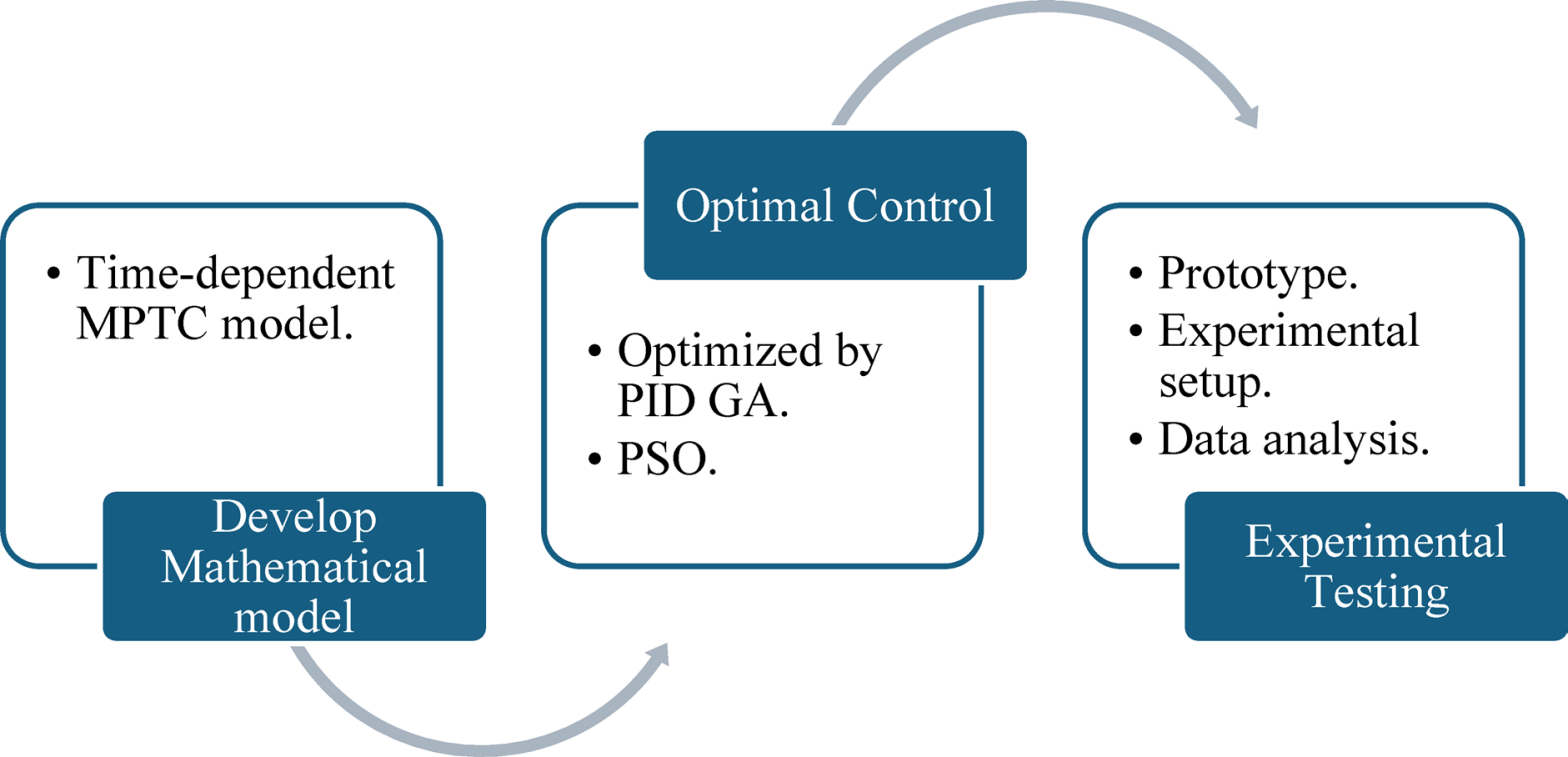
Research article workflow.

This systematic approach integrates theoretical modeling with experimental validation, enabling a comprehensive assessment of the MPTC’s performance. The overall process is outlined in the flowchart shown in Fig. 2.

## Mathematical model

In this section the MPTC concept of operation, in the form of optical, thermal and dynamic models, is discussed along with its mathematical foundation. Two optimized PID controllers, using GA and PSO, are developed and applied to MPTC.

### The optical model

**Fig. 3:**
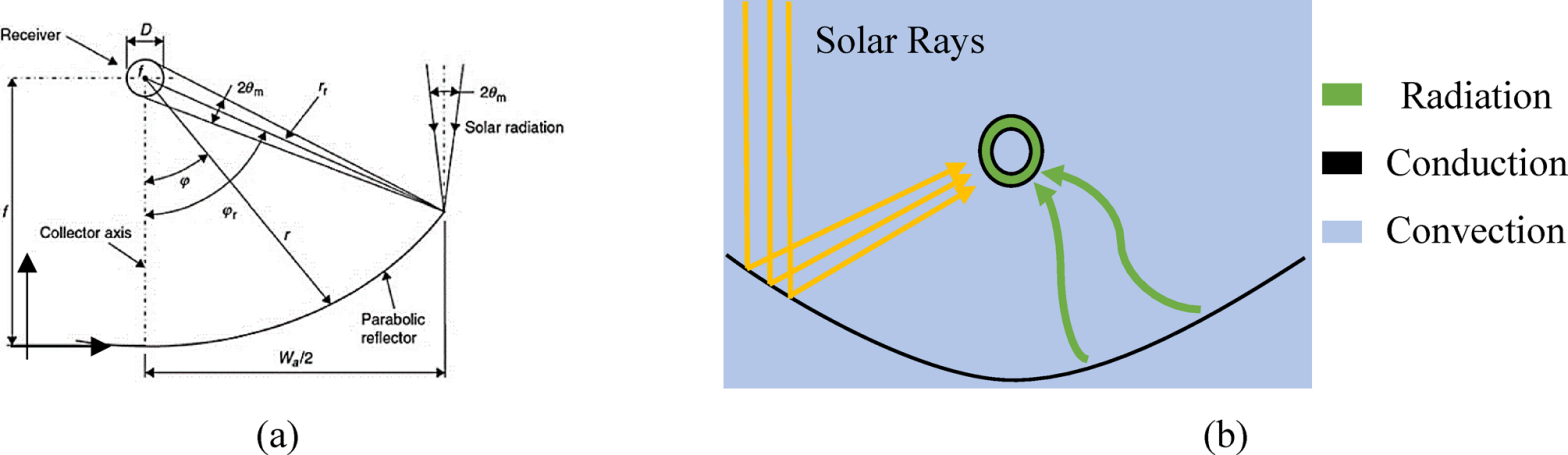
PTC operation parameters: (**a**) Cross-section of a PTC^30,31^, and (**b**) Heat transfer modes through the PTC operation.

**Fig. 4:**
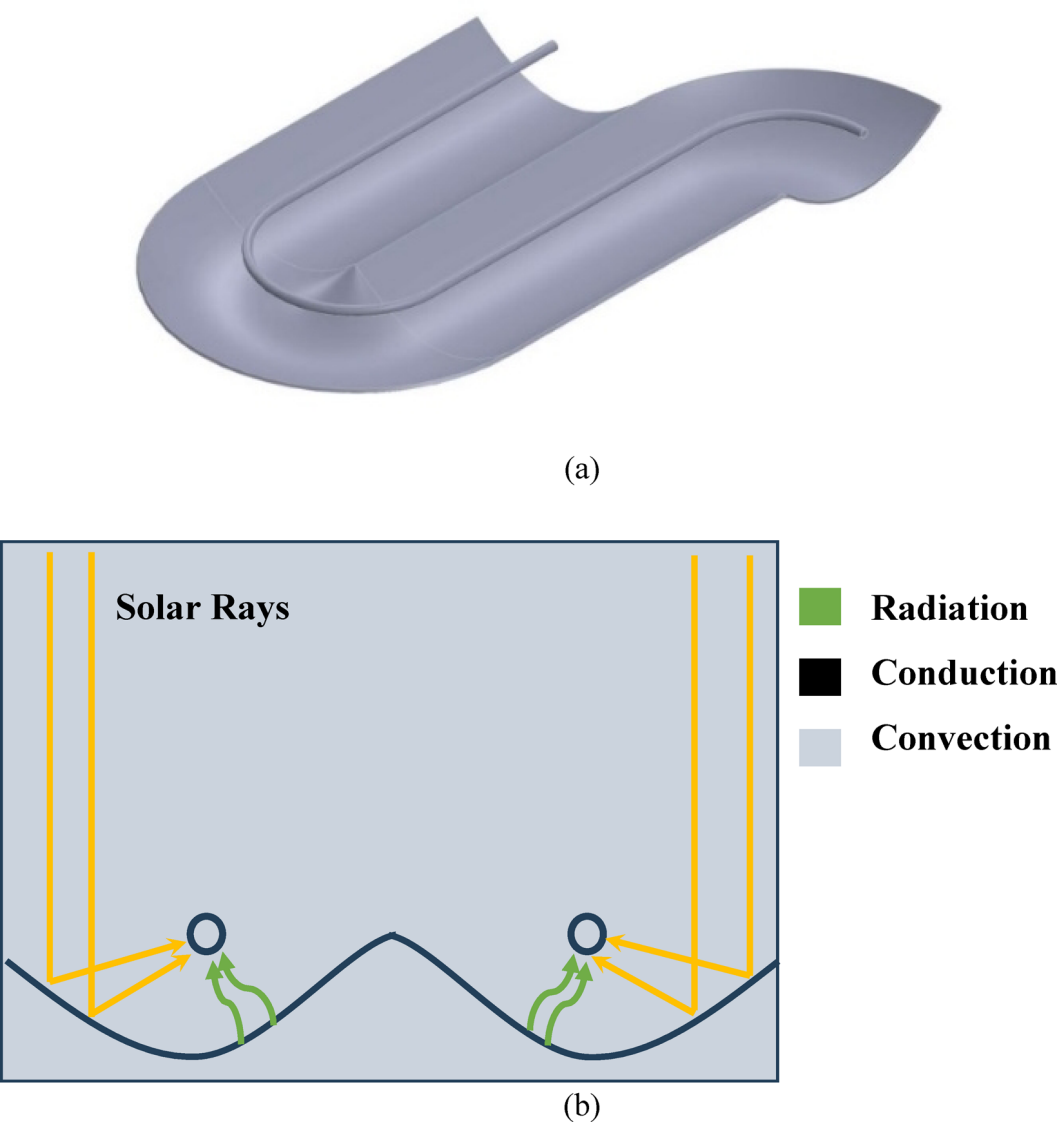
(**a**) MPTC general configuration, created using FreeCAD^32^, and (**b**) MPTC crosssection.

MPTC operates based on the principals of optics and heat transfer, concentrating solar energy collectors onto a receiver (absorber tube) using parabolic reflectors, as shown in Fig. 3. The proposed system incorporates multiple enhanced parabola collectors, as shown in Fig. 4a, to improve energy capture and utilization.

The core concept of the MPTC is to develop a multi-parabola, multi-tube configuration that enhances the performance of conventional PTCs. This design either maintains the same projected aperture area or extends the total length of the absorber tube. As depicted in Fig. 4b, the system connects several smaller parabolic segments within the same footprint, effectively creating a longer absorber tube. This arrangement facilitates cumulative heating, wherein the fluid exiting each stage—at an elevated temperature—serves as the inlet for the subsequent stage, thereby maximizing energy transfer across the system.

A clear understanding of the geometric characteristics and optical efficiency of the system is essential to elucidate the fundamental mechanisms driving its operation. The geometry of the PTC directly influences its optical performance, as it focuses on incoming solar radiation onto the absorber tube, thereby heating the working fluid along the focal line. Semi-analytical models for MPTCs typically consist of mathematical formulations that describe the system’s optical, thermal, and fluid dynamics behavior. These models are derived from a set of nonlinear equations, generally through iterative methods, and are divided into two main sub-models: the optical model and the thermal model (Appendix A).

A customized analytical model is developed for MPTC, specifically tailored to the operating temperature range and properties of pressurized water. The model assumes a diameter-to-length ratio less than or equal to 0.024 and excludes multiphase flow. The modeling process begins with the optical sub-model, which establishes the geometry of the parabolic collector. The shape of the collector is mathematically defined by Eq. (1), forming the foundation for subsequent thermal and optical analyses. This structures approach enables a precise representation of the system’s behavior and supports effective performance prediction and optimization.

1 $$y=\frac{{x}^{2}}{4f}$$

with symmetry about the y-axis, $f$ is the focal length of the parabola and ${w}_{a}$ is the aperture width. The relationship between $f$ and ${w}_{a}$ is defined as^33^:

2 $$\frac{{w}_{a}}{f}=-\frac{4}{\mathit{tan}{\varphi }_{r}}+\sqrt{\frac{16}{\mathit{tan}\left({\varphi }_{r}^{2}\right)}+16}$$

and the parabola height,

3 $${H}_{p}=\frac{W}{4*\mathrm{tan}\frac{{\varphi }_{r}}{2}}$$

4 $${A}_{a}={w}_{a}*L$$

${A}_{a}$ is the aperture area. In the case of MPTC, $L$ denotes the total length of the MPTC^33^.

The rim angle ${\varphi }_{r}$ is expressed as:

5 $${\varphi }_{r}=\frac{{\mathit{sin}}^{-1}{w}_{a}}{2r}$$

The combined losses resulting from geometric imperfections in an optical system and optical qualities of materials, such as absorptivity, emissivity of the absorber tube, reflectivity of the mirror or reflector, and transmissivity of the glass cover, are represented in “Optical Efficiency”: it is a measure of how flawless the system is, and is formally stated as,

6 $${\eta }_{o}={\rho }_{r}\gamma \tau \alpha k\left({\theta }_{m}\right)$$

where ${\rho }_{r}$ denotes the mirror’s reflectance, $\tau$ represents the glass cover’s transmittance, $\alpha$ Indicates the receiver’s absorptance, $\gamma$ refers to the intercept factor, ${\theta }_{m}$ Stands for the angle of incidence, and *k* is an indicator of the inclination angle given by^33^:

7 $$k({\theta }_{m})=\left(\left(1-{A}_{f}*tan\left({\theta }_{m}\right)\right)*cos\left({\theta }_{m}\right)\right)$$

where ${A}_{f}$ is a geometric factor, given by:

8 $${A}_{f}=\frac{{A}_{e}+{A}_{b}}{{A}_{a}}=\frac{\left(f{w}_{a}\mathrm{tan}{\theta }_{m}\left[1+\frac{{w}_{a}^{2}}{48{f}^{2}}\right]\right)+\frac{2}{3}{w}_{a}{H}_{p}\mathrm{tan}{\theta }_{m}}{{A}_{a}}$$

here ${A}_{e}$ is the aperture area lost, ${A}_{b}$ represents the loss of aperture area by the opaque plates to preclude unwanted or dangerous concentration away from the receiver.

The boundary conditions are defined as the inlet temperature ${T}_{in,j}$ and the time-varying ambient temperature ${T}_{am}.$

## Model assumptions and boundary conditions

The boundary and initial conditions in the present model are defined according to the prevailing atmospheric conditions in Egypt, and include the following assumptions:

- Single-phase water flow is considered.
- Solar irradiance and ambient temperature vary, with initial values of ${I}_{c}\left(0\right)=933\frac{W}{{m}^{2}}$, and ${T}_{emb}=300 K$.
- The inlet flow rate is constant and determined by the pump specifications listed in Table 1.

**Table 1:** Experimental setup components and specifications.

Component	Material/number	Dimensions/specifications
Collector	Aluminum sheets bended in parabolic trough form	900*1152 mm
Tube	Copper	Φ15 mm, $\sim$ 4 m long
Pump with drive	1	H = head, 2m, Q = flowrate, 5.655 L/hr, and operating fluid is water Submersible with outlet up to Φ 15 mm
Tank	1	50L equipped with sufficient installation for the inlet and outlet flow from the MPTC itself
Temperature sensor	3	Waterproof Temperature sensors up to 150 °C
Arduino UNO Control kit	1	Microcontroller kit (Arduino Atmega)

### Time-dependent-modeling

**Fig. 5:**
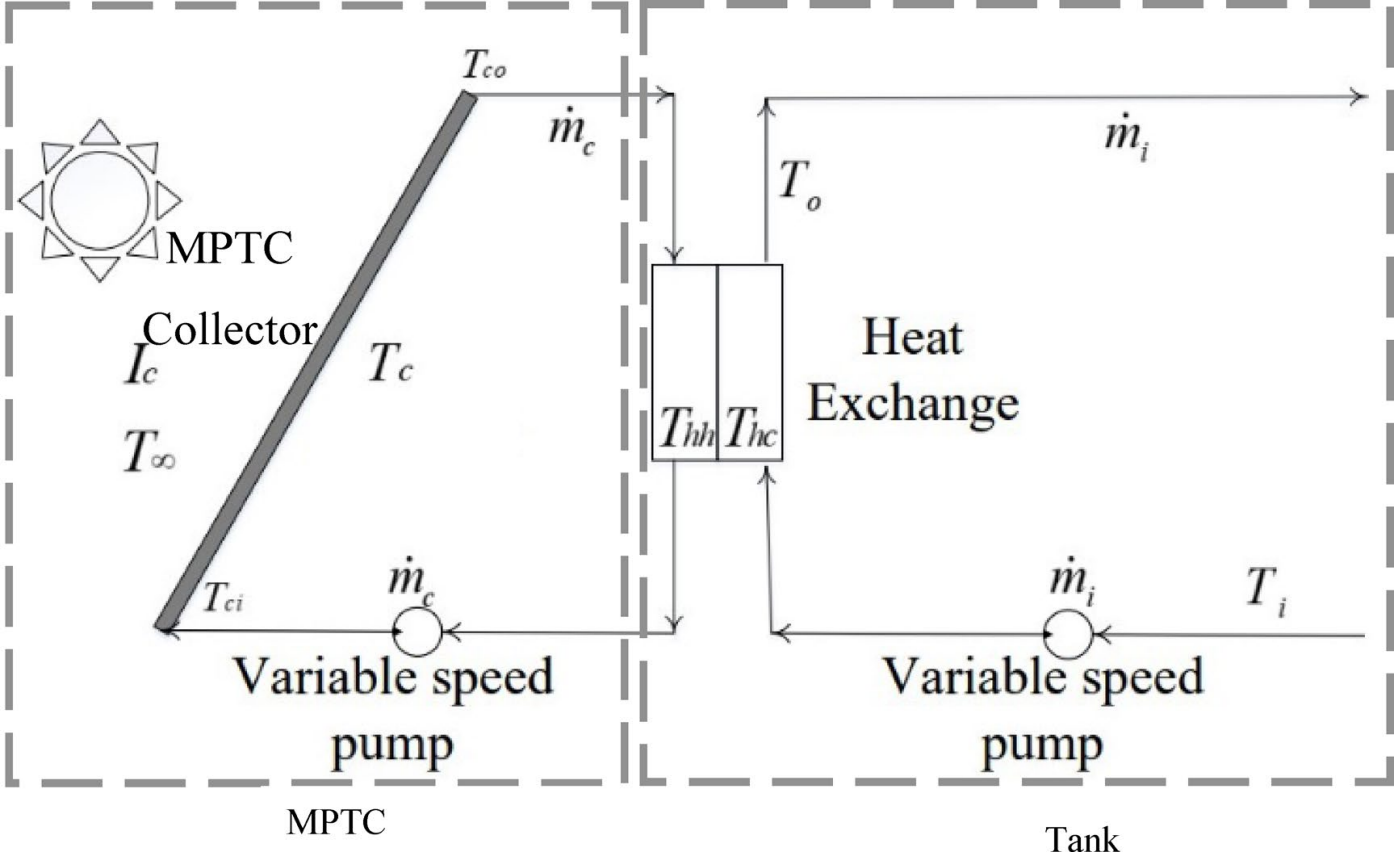
System configuration^2^.

The overall transfer function is separated into two components: the MPTC component and the storage tank component, as shown in Fig. 5. The outlet temperature ${T}_{co}$ is primarily influenced by input variables such as solar irradiance ${I}_{c}$, inlet temperature ${T}_{ci}$, and the ambient temperature ${T}_{\infty }$. Similarly, the storage tank temperature ${T}_{hc}$ depends on solar irradiance, inlet temperature ${T}_{i}$, and the MPTC outlet temperature ${T}_{co}$.

The MPTC energy balance is defined as following^4^:

9 $${Q}_{u}={\eta }_{o}{Q}_{s}-{Q}_{loss}$$

where ${\eta }_{o}$ denotes the optical efficiency, and ${Q}_{loss}$ is the total heat loss due to convection and radiation,

10a $${Q}_{s}={A}_{a} GB\left(t\right)$$

10b $${Q}_{loss}={C}_{c}\frac{d{T}_{co}}{dt}={A}_{co}{h}_{out}\left({T}_{c}- {T}_{\infty }\right)+{A}_{co}\sigma {\varepsilon }_{c}\left({T}_{c}^{4} - {T}_{\infty }^{4}\right)$$

10c $${Q}_{u} = h {A}_{ri} ({T}_{r} - {T}_{co} )$$

10d $${C}_{c}=\rho cV$$

Here ${A}_{a}$ is the aperture area of the solar receiver tube, ${C}_{c}$ is the fluid heat capacity [J/K], $\rho$ is the fluid mass density, $c$ is the fluid specific heat capacity (J/kg K), and $V$ is the fluid volume inside the receiver tube.

Then the outlet temperature rate of change $$can obtained from$$^6^

11 $$\frac{d{T}_{co}\left(t\right)}{dt}=\frac{{\eta }_{o} {A}_{a} {I}_{c}\left(t\right)}{{C}_{p}}-\frac{{U}_{L}{A}_{a}}{{C}_{p}}\left({T}_{\infty }\left(t\right)-{T}_{c}\left(t\right)\right)+\frac{v\left(t\right)}{V}\left({T}_{ci}\left(t\right)-{T}_{co}\left(t\right)\right)$$

12 $${U}_{L}={U}_{L}={\left[\frac{{A}_{r}}{\left({h}_{c,c-a}+{h}_{r,c-a}\right){A}_{g}}+\frac{1}{{h}_{r,r-c}}\right]}^{-1}$$

As ${\rho }_{r}$ denotes the mirror’s reflectance, $\tau$ is the glass cover’s transmittance, $\alpha$ defines the receiver’s absorptance, $\gamma$ is the intercept factor, ${\theta }_{m}$ represents the angle of incidence, *k* is an indicator of the inclination angle, ${A}_{r}$ is the receiver area, ${A}_{g}$ is the area of the glass cover, ${h}_{c,c-a}$ represents the convection coefficient of the losses in the glass cover which can be calculated as ${h}_{c,c-a}=\frac{Nu.k}{{D}_{co}}$, and $Nu$ is the Nusselt number^30–33^.

Similarly, the energy balance in MPTC with the storage tank system^34^:

13 $$\frac{d{Q}_{tan{k}_{hc}}}{dt}={Q}_{u}-{Q}_{loss}-{Q}_{load}$$

Assuming that there is no consumption load ${Q}_{load}=0$

14 $$\frac{d{Q}_{tan{k}_{hc}}}{dt}={Q}_{u}-{Q}_{loss}$$

Based on the energy balance in the solar heating system, including the storage tank, the rate of change of the storage temperature, $\frac{d{T}_{hc}\left(t\right)}{dt}$, is expressed as follows^7^:

15a $$\frac{d{T}_{hc}\left(t\right)}{dt}=\frac{{\rho }_{i}{C}_{i}{v}_{i}}{\frac{{c}_{h}{m}_{h}}{2}+\frac{{\rho }_{i}{C}_{i}{V}_{h}}{2}}\left({T}_{i}\left(t\right)-{T}_{hc}\left(t\right)\right)+\frac{\varepsilon {K}_{h}{A}_{h}}{\frac{{c}_{h}{m}_{h}}{2}+\frac{{\rho }_{i}{C}_{i}{V}_{h}}{2}}\left({T}_{hh}\left(t\right)-{T}_{hc}\left(t\right)\right)+\frac{{A}_{he}{K}_{he}}{\frac{{c}_{h}{m}_{h}}{2}+\frac{{\rho }_{i}{C}_{i}{V}_{h}}{2}}\left({T}_{co}\left(t\right)-{T}_{hc}\left(t\right)\right)$$

15b $$\frac{d{T}_{c}\left(t\right)}{dt}=\frac{{\eta }_{o} {A}_{a} GB\left(t\right)}{{C}_{p}}-\frac{{U}_{L}{A}_{a}}{{C}_{p}}\left({T}_{\infty }\left(t\right)-{T}_{c}\left(t\right)\right)+\frac{v\left(t\right)}{V}\left({T}_{hh}\left(t\right)-{T}_{c}\left(t\right)\right)$$

15c $$\frac{d{T}_{hh}\left(t\right)}{dt}=\frac{{\rho }_{c}{C}_{c}{v}_{c}}{\frac{{c}_{h}{m}_{h}}{2}+\frac{{\rho }_{c}{C}_{c}{V}_{h}}{2}}\left({T}_{c}\left(t\right)-{T}_{hh}\left(t\right)\right)+\frac{\varepsilon {K}_{h}{A}_{h}}{\frac{{c}_{h}{m}_{h}}{2}+\frac{{\rho }_{c}{C}_{c}{V}_{h}}{2}}\left({T}_{hc}\left(t\right)-{T}_{hh}\left(t\right)\right)+\frac{{A}_{he}{K}_{he}}{{c}_{h}{m}_{h}+{\rho }_{c}{C}_{c}{V}_{h}}\left({T}_{co}\left(t\right)-{T}_{hh}\left(t\right)\right)$$

where ${U}_{L}$ denotes the heat loss coefficient, ${C}_{p}$ is the heat capacity of the HTF, $v$ is the fluid volume flow rate, and $V$ defines the volume of the fluid in the receiver tube.

### Closed loop system control

The increasing demand for a robust and systematic control architecture is driven by the need to maximize the efficiency of the MPTC, suppress external disturbances, and ensure operational stability^7^. The proposed control strategy is designed to maintain a constant storage tank temperature under varying solar irradiance and ambient conditions^6^. In this context, a proportional–integral–derivative (PID) controller is developed to regulate the storage tank temperature by modulating the flow rate of the working fluid. The PID controller is adopted due to its simplicity, reliability, and demonstrated effectiveness in minimizing steady-state errors^35,36^.

**Fig. 6:**
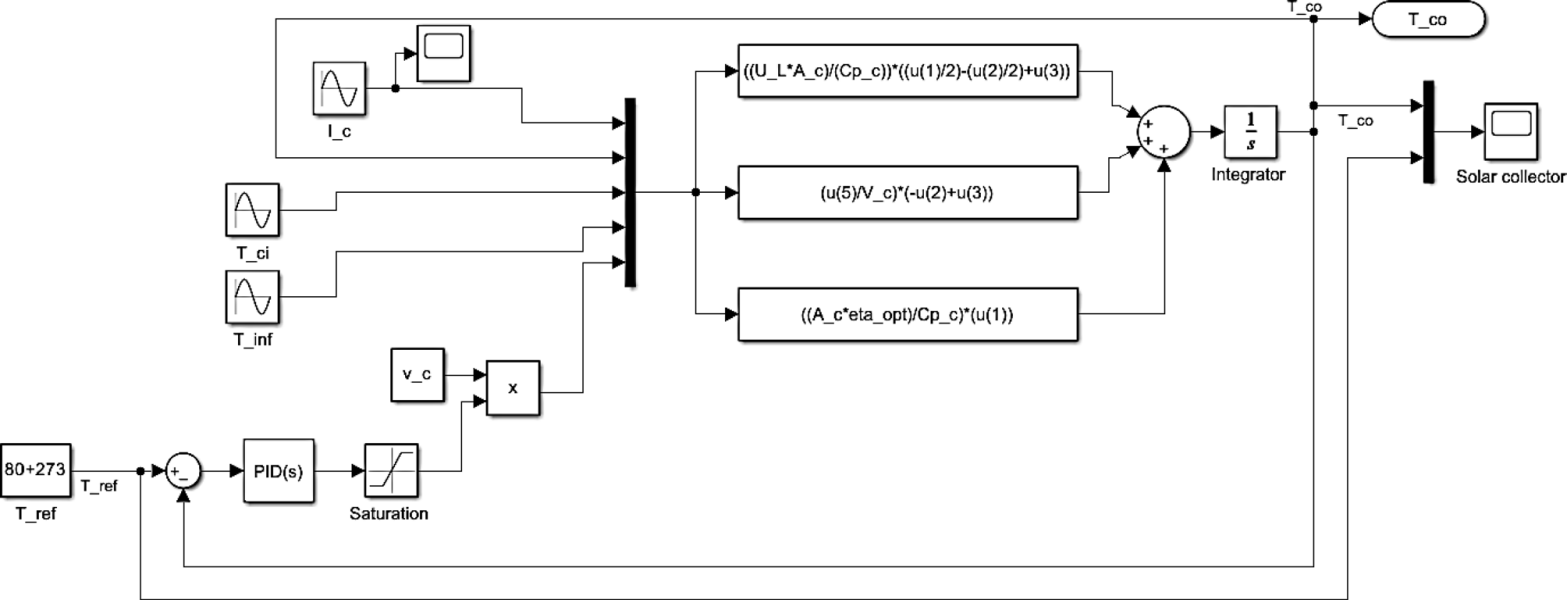
MPTC Simulink model.

Figure 6 illustrates the SIMULINK model for the novel MPTC.

The MPTC and storage tank models are linearized around a designated operational point to facilitate the use of transfer functions corresponding to each input variable. The volume flow rate, $v$, serves as the manipulated variable to regulate the storage tank temperature, ${T}_{hc}\left(t\right)$. Two closed-loop PID controllers are implemented: one for the standalone MPTC model and another for the MPTC integrated with storage tank. Each controller is characterized by the proportional gain (${K}_{p}$), integral gain (${K}_{i}$), and derivative gain (${K}_{d}$), with subscripts “MPTC” for the standalone model (Case 01) and MPTC_tank_ for the integrated system (Case 02), as defined in the controller equation below:

16 $$v(t)={K}_{p}e\left(t\right)+{K}_{i}{\int }_{0}^{t}e\left(t\right)dt+{K}_{d}\frac{d}{dt}e\left(t\right)$$

where in the case of the MPTC model

17 $$e\left(t\right)={T}_{{co}_{ref}}-{T}_{co}\left(t\right)$$

and in the case of the MPTC integrated with tank model

$$e\left(t\right)={T}_{{hc}_{ref}}-{T}_{hc}\left(t\right)$$

As ${T}_{{co}_{ref}}$ and ${T}_{h{c}_{re}f}$ denote the desired MPTC outlet temperature and desired storage temperature, respectively.

**Fig. 7:**
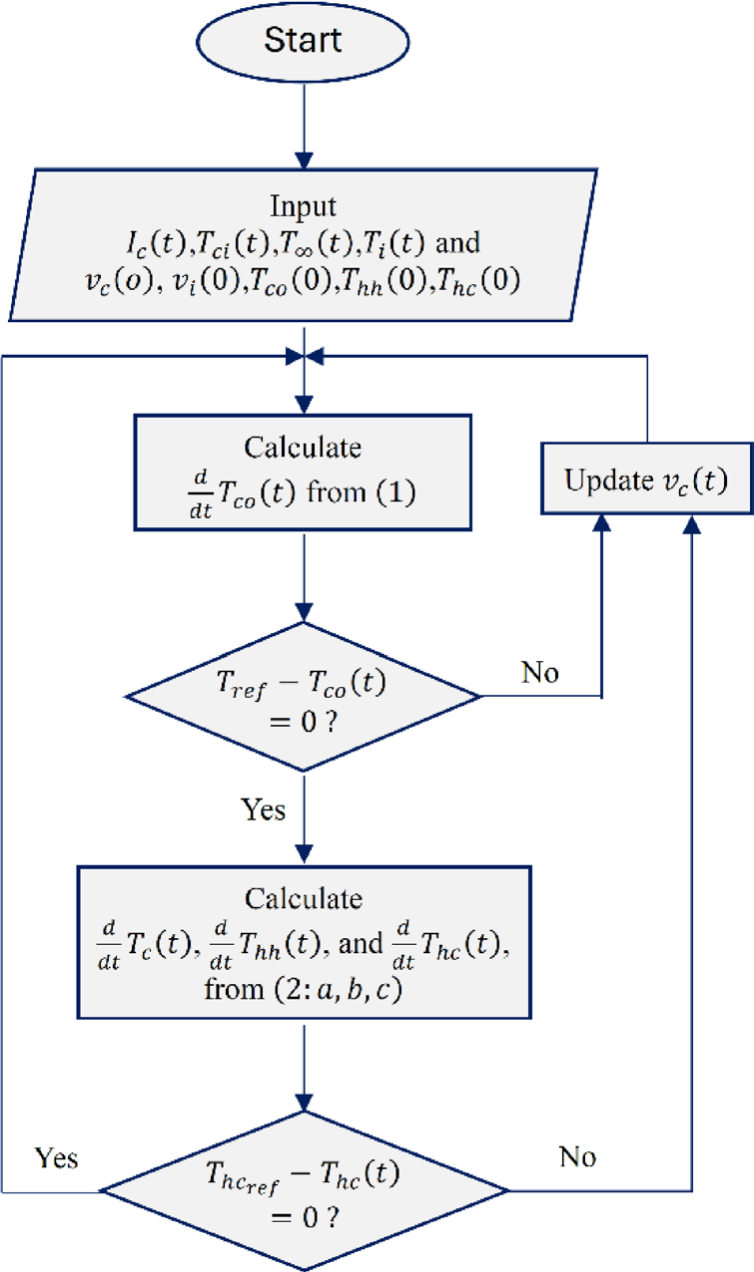
Flowchart of the MPTC model combined with storage tank system Simulink model.

The system response to the desired temperature,${T}_{h{c}_{re}f}$, is analyzed within a closed loop transfer function, as follows:

18 $${W}_{cl}\left(s\right)=\frac{{W}_{c}\left(s\right){W}_{out}\left(s\right)}{1+{W}_{c}\left(s\right){W}_{out}\left(s\right)}$$

where ${W}_{cl}\left(s\right), {W}_{c}\left(s\right)$, and ${W}_{out}\left(s\right)$ represent the transfer functions of the closed-loop system, the controller, and the system output, respectively. Figure 7 illustrates the flowchart of the complete MPTC model integrated with the storage tank.

### Optimization

In this study, both GA and PSO algorithms are employed to optimize the PID controller for the two developed cases. The design objectives are defined as to minimize the errors between the desired and achieved output temperatures. The results can help us to compare between the two optimization methods. The optimization problem for Case 01 is defined as:

Find $$\mathbf{x}= \left[{k}_{{p}_{\mathrm{MPTC}}}, {k}_{{i}_{\mathrm{MPTC}}}, {k}_{{d}_{\mathrm{MPTC}}}\right]$$

That minimize:

19 $$\begin{aligned} & {\boldsymbol{e}}_{{{\boldsymbol{MPTC}}}} \left( t \right) = 0.25{*}\sqrt {\sum \left( {T_{co} - T_{{cohc_{ref} }} } \right)^{2} + \sum \left| {\left( {T_{co} - T_{{hc_{ref} }} } \right)_{ } } \right| + \sum \left| {\left( {T_{co} - T_{{co_{ref} }} } \right)_{ } } \right|*t + \sum \left( {T_{co} - T_{{co_{ref} }} } \right)^{2} *t} \\ & {\mathrm{Subject}}\;{\mathrm{to}}\;0 \le {\boldsymbol{Error}} < 10^{ - 5} \\ \end{aligned}$$

where the MPTC controller equation is as follows:

20 $${v}_{MPTC}(t)={K}_{{p}_{\mathrm{MPTC}}}{e}_{{\boldsymbol{M}}{\boldsymbol{P}}{\boldsymbol{T}}{\boldsymbol{C}}}\left(t\right)+{K}_{{i}_{\mathrm{MPTC}}}{\int }_{0}^{t}{e}_{{\boldsymbol{M}}{\boldsymbol{P}}{\boldsymbol{T}}{\boldsymbol{C}}}\left(t\right)dt+{K}_{{d}_{\mathrm{MPTC}}}\frac{d}{dt}{e}_{{\boldsymbol{M}}{\boldsymbol{P}}{\boldsymbol{T}}{\boldsymbol{C}}}\left(t\right)$$

whereas, the optimization problem for the PID controller for Case 02 is defined as:

Find $$\mathbf{x}= \left[{k}_{{p}_{{\mathrm{MPTC}}_{tank}}}, {k}_{{i}_{{\mathrm{MPTC}}_{tank}}}, {k}_{{d}_{{\mathrm{MPTC}}_{tank}}}\right]$$

That minimize:

21 $$\begin{aligned} & {\boldsymbol{e}}_{{{\mathrm{MPTC}}_{tank} }} \left( t \right) = 0.25{*}\sqrt {\sum \left( {T_{hc} - T_{{hc_{ref} }} } \right)^{2} + \sum \left| {\left( {T_{hc} - T_{{hc_{ref} }} } \right)} \right| + \sum \left| {\left( {T_{hc} - T_{{hc_{ref} }} } \right)} \right|*t + \sum \left( {T_{hc} - T_{{hc_{ref} }} } \right)^{2} *t} \\ & {\text{Subject to}}:0 \le {\boldsymbol{e}}_{{{\mathrm{MPTC}}_{tank} }} < 10^{ - 5} \\ \end{aligned}$$

where the tank controller equation is as follows:

22 $${v}_{{\mathrm{MPTC}}_{tank}}(t)={K}_{{p}_{{\mathrm{MPTC}}_{tank}}}{{\boldsymbol{e}}}_{{\mathrm{MPTC}}_{tank}}\left(t\right)+{K}_{{i}_{{\mathrm{MPTC}}_{tank}}}{\int }_{0}^{t}{{\boldsymbol{e}}}_{{\mathrm{MPTC}}_{tank}}\left(t\right)dt+{K}_{{d}_{{\mathrm{MPTC}}_{tank}}}\frac{d}{dt}{{\boldsymbol{e}}}_{{\mathrm{MPTC}}_{tank}}\left(t\right)$$

**Fig. 8:**
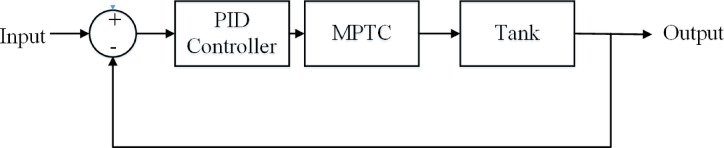
System block diagram.

Figure 8 shows the block diagram of the system model.

#### Genetic algorithm

Genetic Algorithm (GA) is an optimization technique inspired by the principles of natural selection. In this study, GA is used to optimize the PID controller parameters with the objective of minimizing control system error.As a population-based search method, GA leverages the concept of 'survival of the fittest’ to iteratively evolve and refine candidate solutions toward optimal performance^37^.

#### Particle swarm optimization

Particle Swarm Optimization (PSO) is an optimization algorithm inspired by the collective behavior of bird flocking and fish schooling. It represents a population of candidate solutions, known as particles, which learn from both their own best-known positions and the best-known positions within the swarm. Each particle adjusts its trajectory based on personal experience and the swarm’s collective knowledge. This iterative process enables PSO to efficiently explore complex and high-dimensional search spaces to identify high-quality solutions, with both complex and high-dimensional problems efficiently. Due to its simplicity and effectiveness, PSO is widely applied in control system optimization, particularly in challenging environments such as solar heating applications. The flowchart of the PSO algorithm is illustrated in Fig. 6^16^.

## Experimental setup

**Fig. 9:**
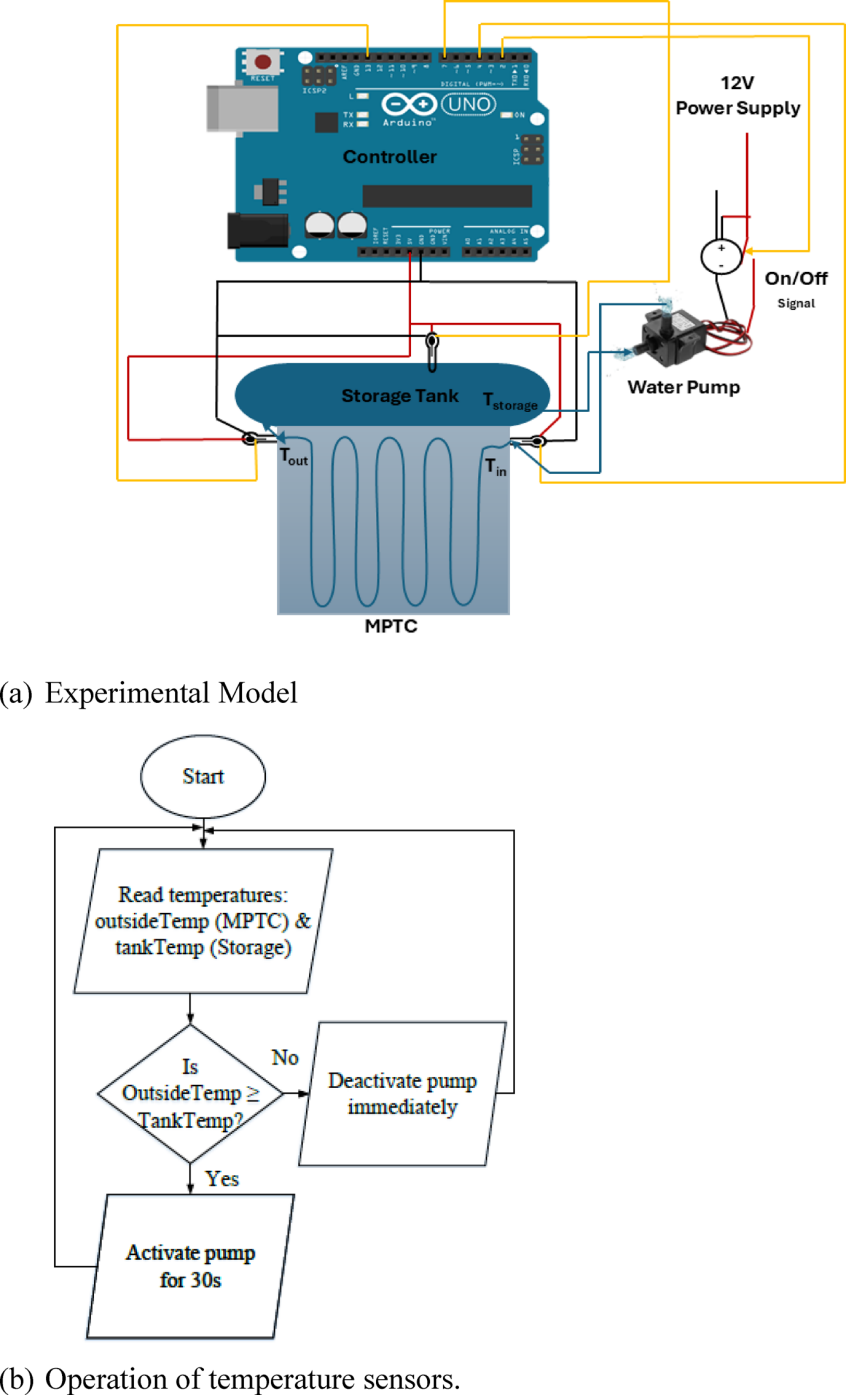
The scheme of the experimental setup.

**Fig. 10:**
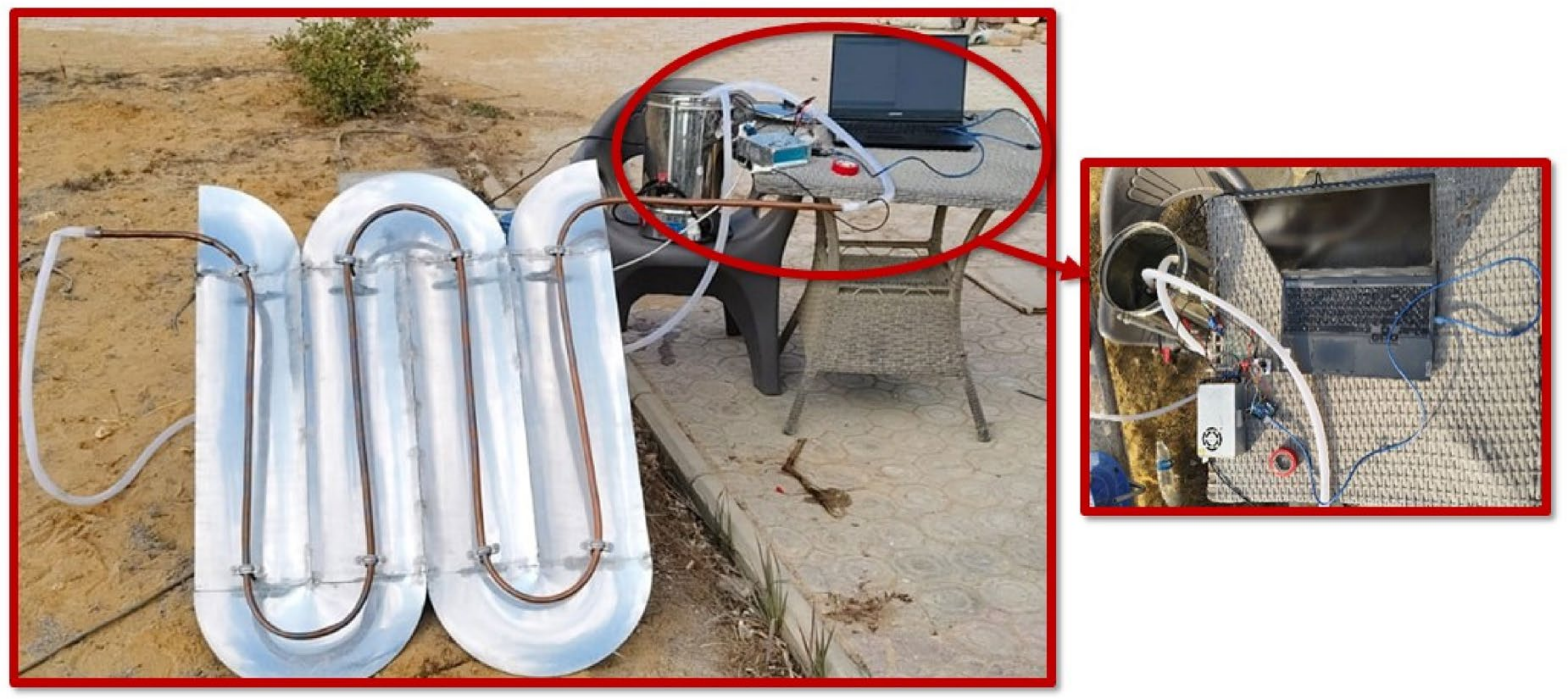
Experimental setup.

The experimental setup involves the testing of the MPTC model. The setup is designed to assess the performance and control capabilities of the solar heater. The system key components are listed in Table 1. The system consists of a collector (reflector), absorber (receiver tube), thermal storage tank, and pump with drive to be controlled according to the sensors readings of temperature. Figure 9 illustrates the experimental setup scheme and Fig. 10 shows the experimental implementation. The experiments are conducted at Nile University, Shiekh Zayed, Giza, Egypt, and was held during between December 2024 and January 2025.

## Model validation

### MPTC system

MPTC, combined with the storage tank, is validated against the models proposed in^6,7^ to verify the accuracy and effectiveness of the proposed design and the control strategy. The validation assesses thermal performance, stability, and the system response to disturbances caused by environmental and climatic conditions. The system accuracy is measured by comparing the tank temperature, volume flow rates, response time, and thermal efficiency of the developed model with similar results from both literature and experiment.

The experimental validation is conducted under conditions where $${T}_{i}=23$$, $${T}_{\infty }=23^\circ{\rm C}$$, and solar irradiance follows a sinusoidal profile with a bias of $${I}_{c}\left(0\right)=933\frac{\mathrm{W}}{{\mathrm{m}}^{2}} ,an initial gradiant of\frac{1}{7200}\frac{\mathrm{W}}{{\mathrm{m}}^{2}\mathrm{s}}$$, and a simulation duration of 7 h , from 10am to 17 pm.

**Fig. 11:**
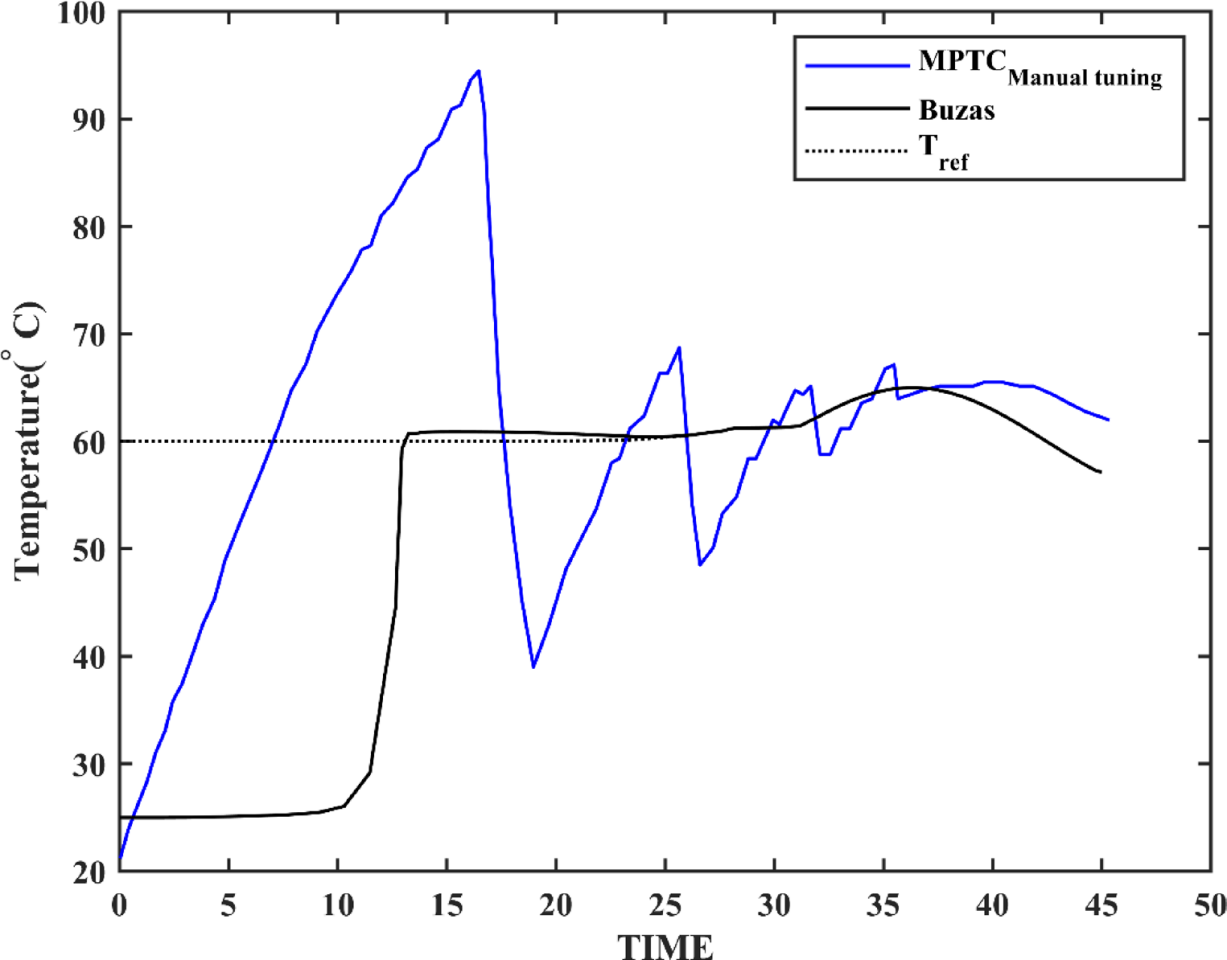
MPTC response versus Buzás model under environmental conditions listed in^6^.

Table 2 compares the performance metrics of the developed MPTC model with those of models from literature. Key performance indicators (KPIs) assessed include settling time, steady-state error, thermal efficiency and temperature difference (ΔT), as listed in Table 2 and illustrated in Fig. 11. The results demonstrate that the developed MPTC outperforms the other models in literature, particularly in terms of faster settling times and better values for both efficiency and output temperature. This improved performance suggests the effectiveness of the control strategies and system dynamics in the present design. However, a slight increase in steady-state error for the basic MPTC model may indicate a trade-off between rapid response and final accuracy.

**Table 2:** MPTC present model validation compared to literature.

	MPTC present model	Buzás et al.^6^
Settling time	10 min	39 min
Steady state error	3.7%	2%
$\Delta T$	42.22 $^\circ{\rm C}$	41.2 $^\circ{\rm C}$
${\eta }_{th}$	16.19%	15.8%

Before the optimization, the PID controller is manually tuned based on the following criteria: achieving critical damping, minimizing settling time, and enhancing disturbance rejection. Table 3 presents the parameters of the manually tuned controller.

**Table 3:** PID controller manual tuning.

PID control gains	Manual tuning
${K}_{p}$	− 500
${K}_{i}$	− 26
${K}_{d}$	0.04

### MPTC_Tank_ system

**Fig. 12:**
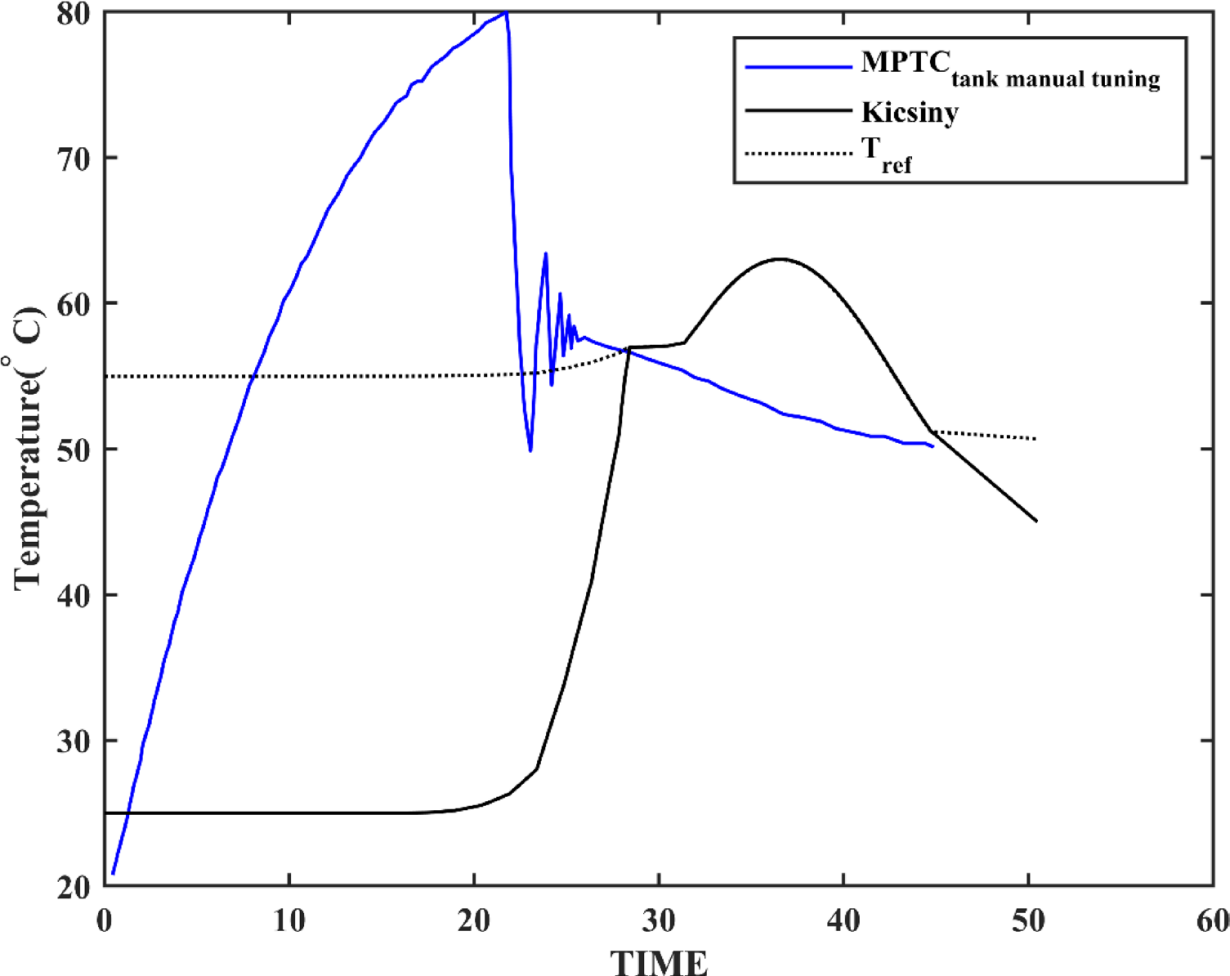
MPTC_tank_ versus Kicsincy model under environmental conditions listed in^7^.

Table 4 compares the performance metrics of the MPTC_Tank_ mathematical model with similar models from literature. The MPTC_Tank_ model demonstrates a significantly improved steady-state error, as shown in Fig. 12. The present MPTC model provides better settling time and steady state errors. The enhanced performance, particularly the faster settling times, suggests more effective control strategies or improved system dynamics in the developed models. The improvement observed in the MPTC_Tank_ model highlights the advantages of incorporating thermal storage, which contributes to greater system stability and precision.

**Table 4:** MPTC with storage tank present model validation compared to literature.

	MPTC with storage tank present model	Kicsiny^7^
Settling time	13.75 min	25 min
Steady state error	0.09%	5%
$\Delta T$	39.9505 $^\circ{\rm C}$	43 $^\circ{\rm C}$

**Fig. 13:**
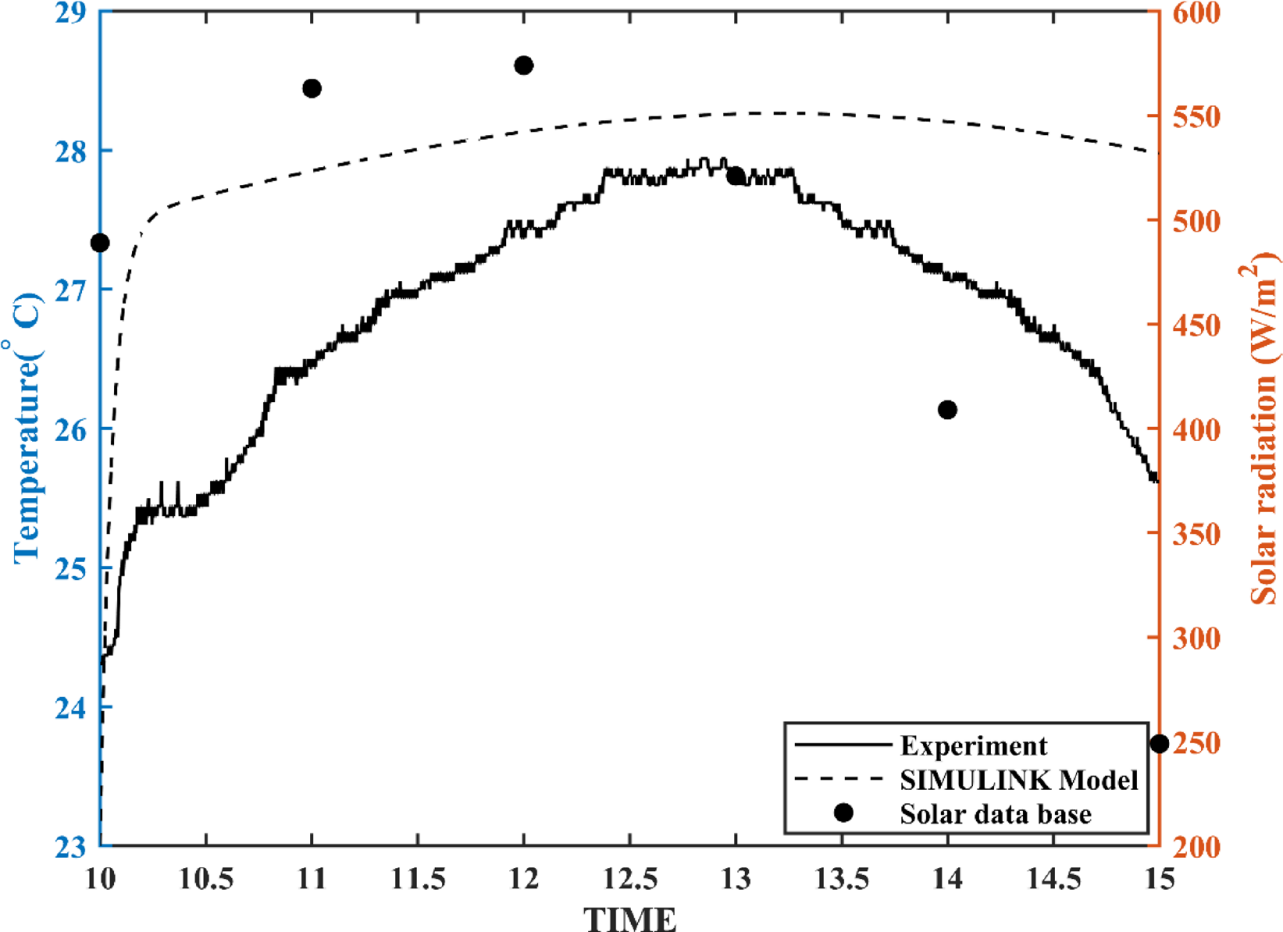
Temperature pattern in response with the solar radiation during the day on 14th November 2024.

A simple model of the receiver tube, without the collector, is tested with a water flow rate of 4 L per minute. A temperature sensor and an Arduino Nano kit are used to record and validate the sensor readings. The recorded data show a correlation with the solar radiation pattern for the same day in November 2024, as obtained from the weather forecast dataset^38^. This correlation is illustrated in Fig. 13.

## Results and discussion

**Fig. 14:**
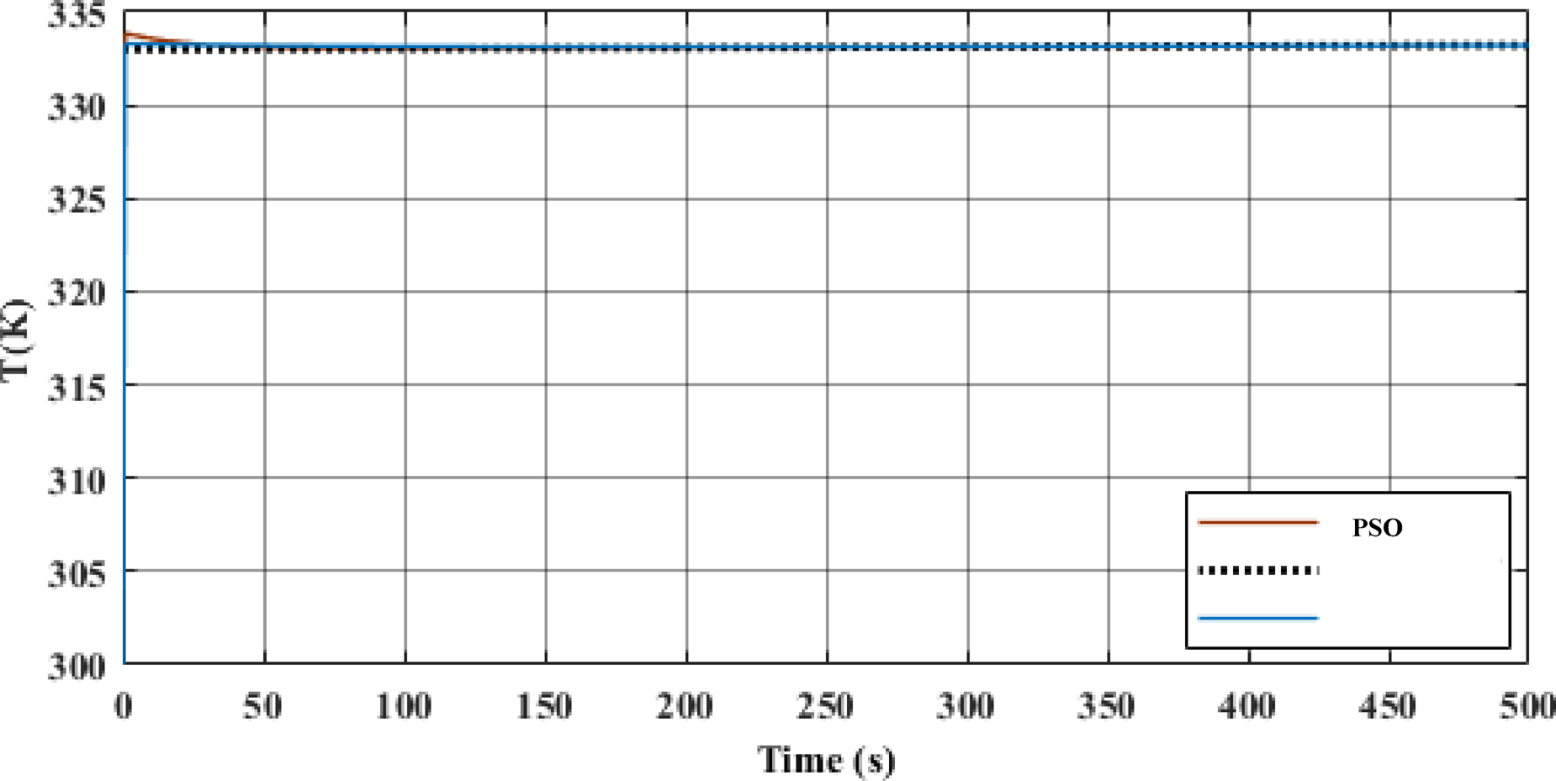
MPTC temperature response under GA (blue) and PSO (red) control under solar irradiance 930 W/m^2^, wind speed 5 m/s, T_am_ 300 K, and measurement uncertainty: ± 0.5 °C.

**Fig. 15:**
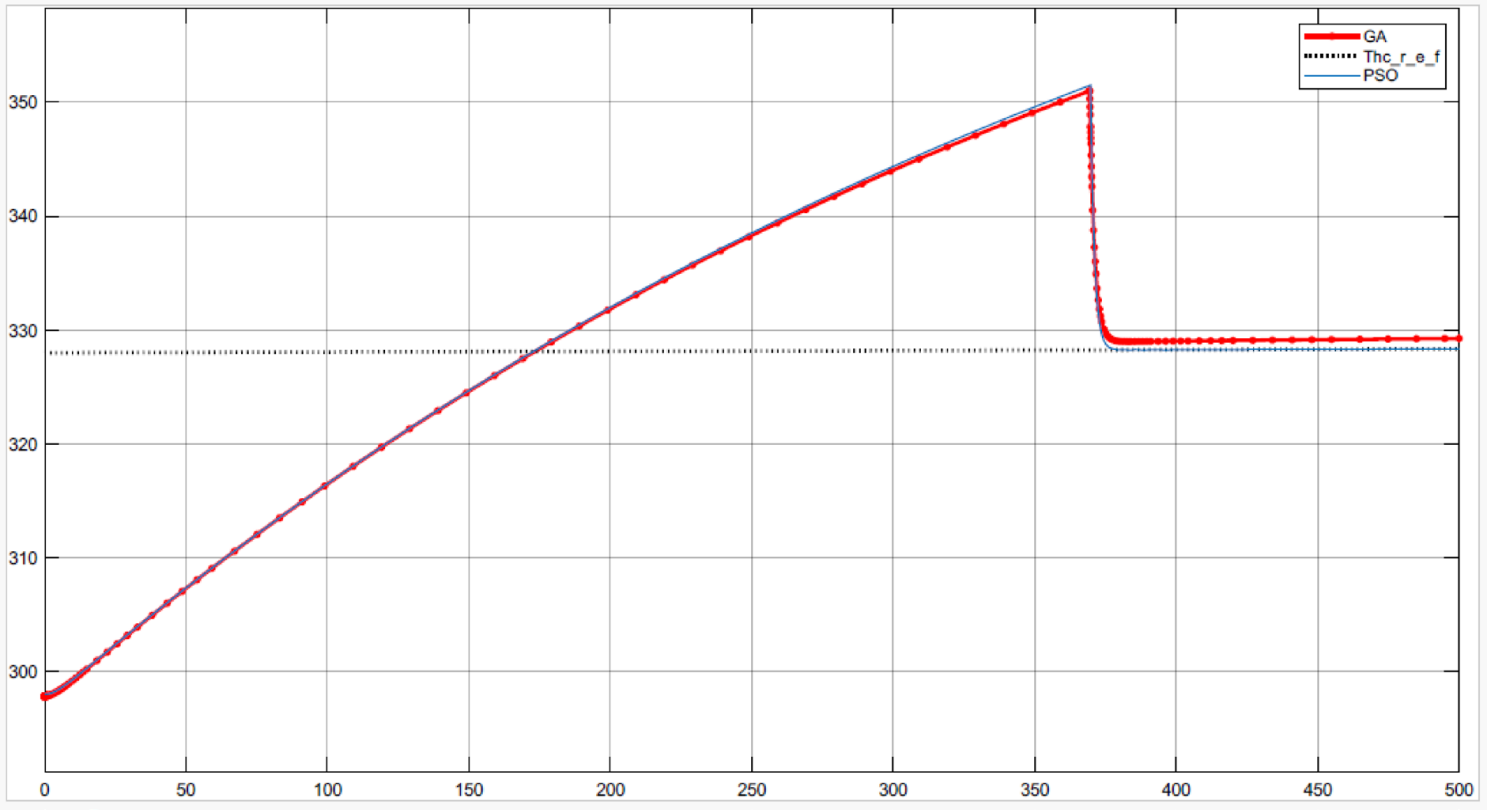
MPTC_Tank_ temperature response under GA (red) and PSO (blue) the two responses are almost identical, under solar irradiance 930 W/m^2^, wind speed 5 m/s, T_am_ 300 K, and measurement uncertainty: ± 0.5.

Table 5, Figs. 14 and 15 compare the GA and PSO results for the PID controller parameters for both MPTC and MPTC_Tank_ with an environmental conditions adopted from^6,7^. The manually tuned PIDs achieved rapid settling time in comparison with the optimized GA and PSO. However, it resulted in very high overshot up to 58.3% in case of MPTC and up to 45.45% in the case of MPTC_Tank_, urging for controller parameters optimization. The differences in the control parameters for the MPTC indicate that PSO produces a more aggressive controller, particularly with a higher ${K}_{p}$ compared to GA. This suggests that the PSO controller may provide a faster response, although it could potentially result in larger oscillations or overshoot, depending on the system dynamics.

**Table 5:** Optimized PID controller parameters.

Controler parameters	Manual tunning	GA	PSO
For the MPTC			
${K}_{p}$	− 500	− 382.1926	− 544.1949
${K}_{i}$	− 26	− 16.9618	− 5.236
${K}_{d}$	0.04	0.0091	0.5437
Settling time	10 min	13 min	14 min
Steady state error	3.7%	0.0000425%	− 0.000085%
Overshoot	58.3%	20%	1.983%
For the MPTC_Tank_			
${K}_{p}$	− 200	− 126.8105	− 108.7209
${K}_{i}$	0.0006	− 75.5192	− 77.3235
${K}_{d}$	0.5	0.6794	0.0308
Settling time	13.75 min	50 min	50 min
Steady state error	0.09%	11%	0.0000024%
Overshoot	45.45%	12%	13.3%
Key advantage	Fast response	Precision	Balanced performance

In terms of settling time, the GA-optimized controller achieves a slightly faster settling time of 13 min, compared to 14 min with PSO. Furthermore, The GA controller’s steady-state error of 0.0000425% translates to a temperature deviation of < 0.01 °C. Conversely, PSO’s 0.0014% error (~ 0.03 °C) remains acceptable for most household systems. While GA achieves superior precision in the MPTC (0.0000425% error), PSO’s faster rise time (Fig. 14) makes it preferable for applications prioritizing rapid heat delivery over absolute accuracy. For the MPTC with Tank, PSO’s 50-min settling time matches GA but with 13.3% overshoot (vs. GA’s 12%), suggesting near-equivalent performance. The choice between algorithms should consider trade-offs: GA for precision, PSO for responsiveness.

For MPTC_Tank_, the control gains optimized by GA and PSO are similar, with only minor variations. Both methods result in negative K_p_ and K_i_ values, which is typical for systems involving thermal storage tanks. However, there is more variation in the derivative gain (K_d_), with GA optimizing a higher value than PSO. This could allow GA to provide more responsive adjustments to dynamic disturbances, particularly in managing the thermal behaviour of the storage tank.

The system employs a real-time flow control strategy based on temperature comparison between the MPTC outlet and the storage tank. When the outlet temperature exceeds the tank temperature, the pump activates for a fixed 30 s to circulate the working fluid and enhance heat transfer. If the tank becomes warmer than the outlet—indicating sunset or cloud cover—the pump shuts off to prevent heat loss. Temperature readings from DS18B20 sensors (updated every 5 s) ensure a responsive control loop that maximizes energy capture while minimizing thermal losses and short cycling.

**Fig. 16:**
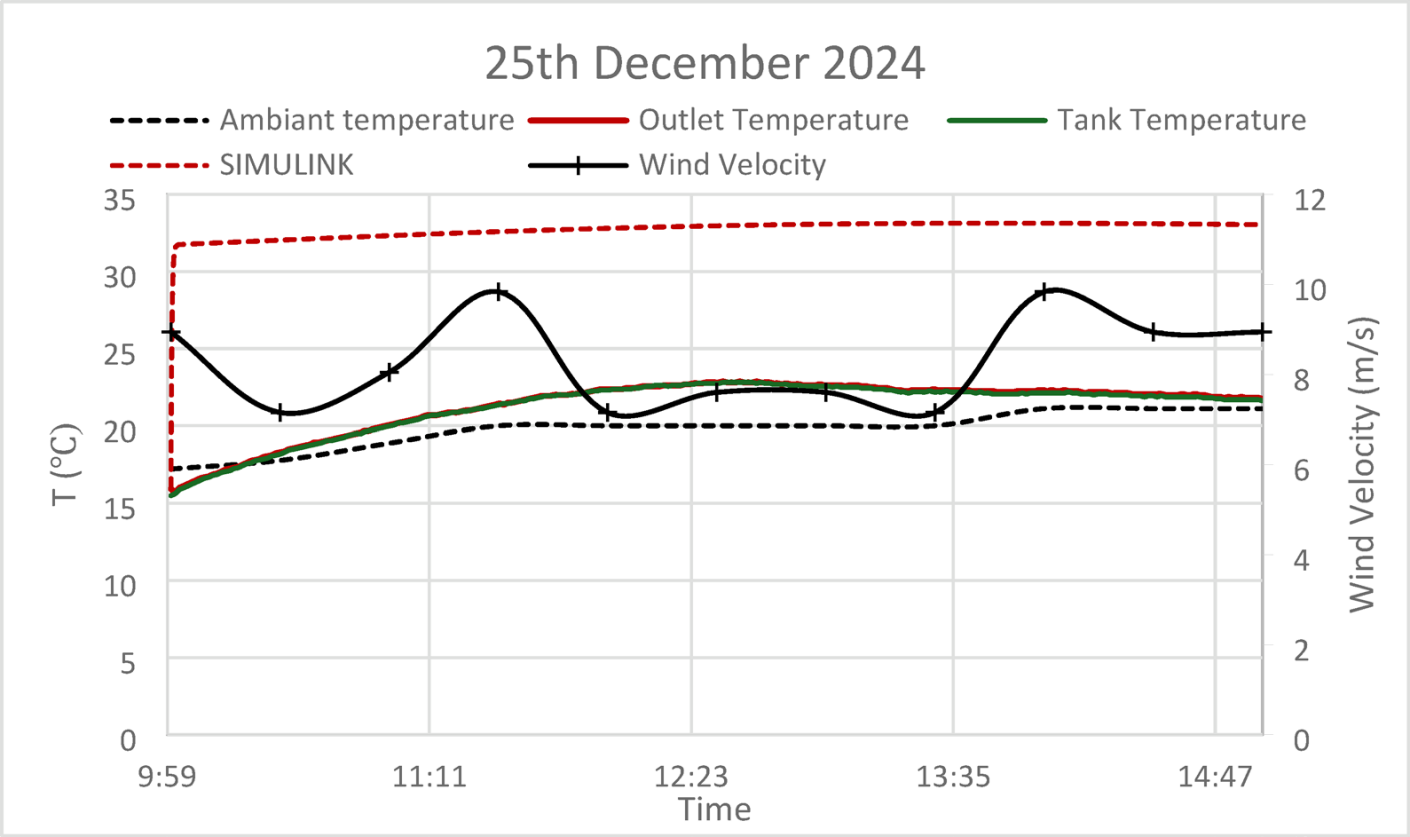
Experimental versus simulink results on 25th December 2024, solar irradiance 930 W/m^2^, wind speed 5 m/s, T_am_ 300 K, and measurement uncertainty: ± 0.5.

**Fig. 17:**
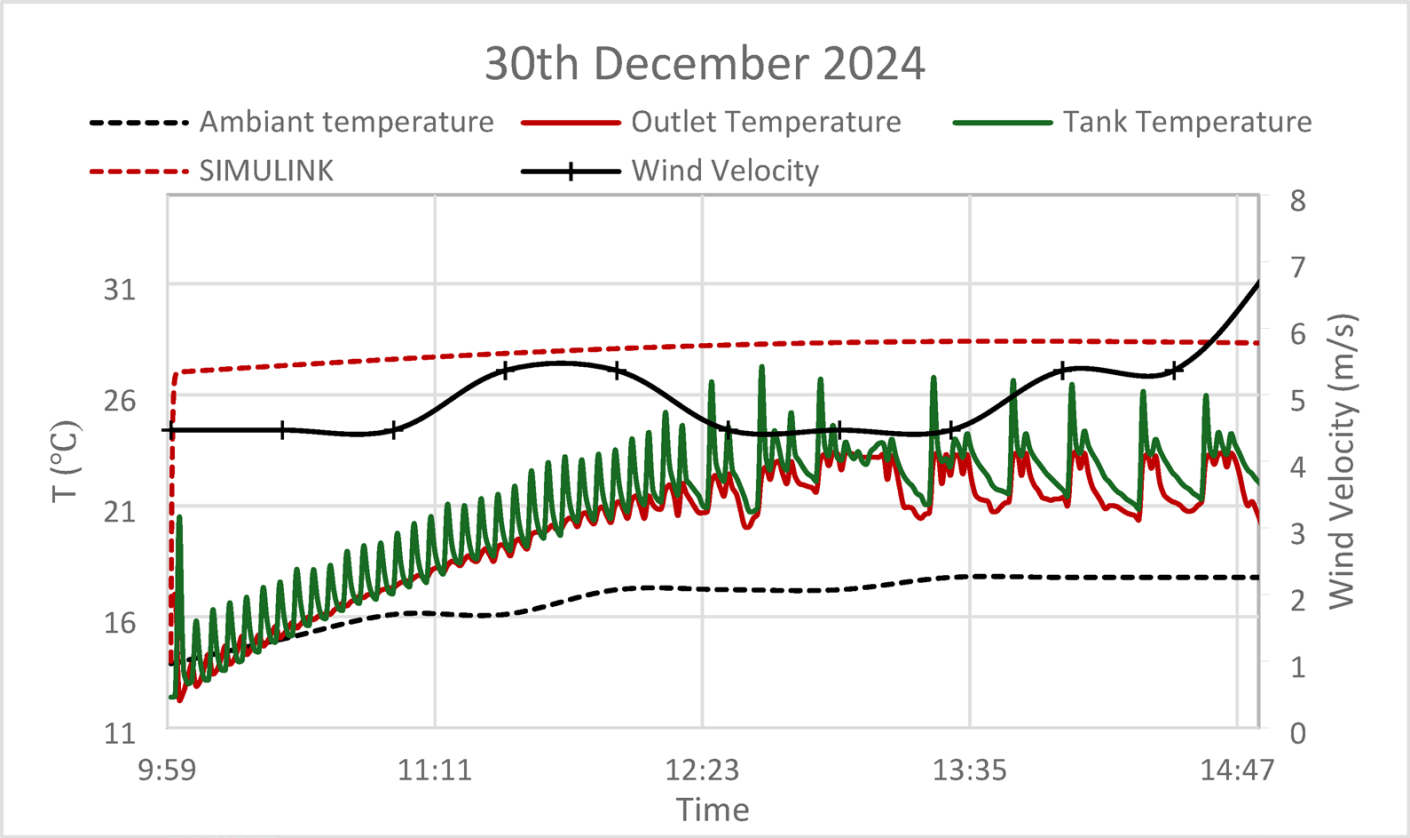
Experimental versus simulink results on 30th December 2024, solar irradiance 930 W/m^2^, wind speed 5 m/s, T_am_ 300 K, and measurement uncertainty: ± 0.5.

**Fig. 18:**
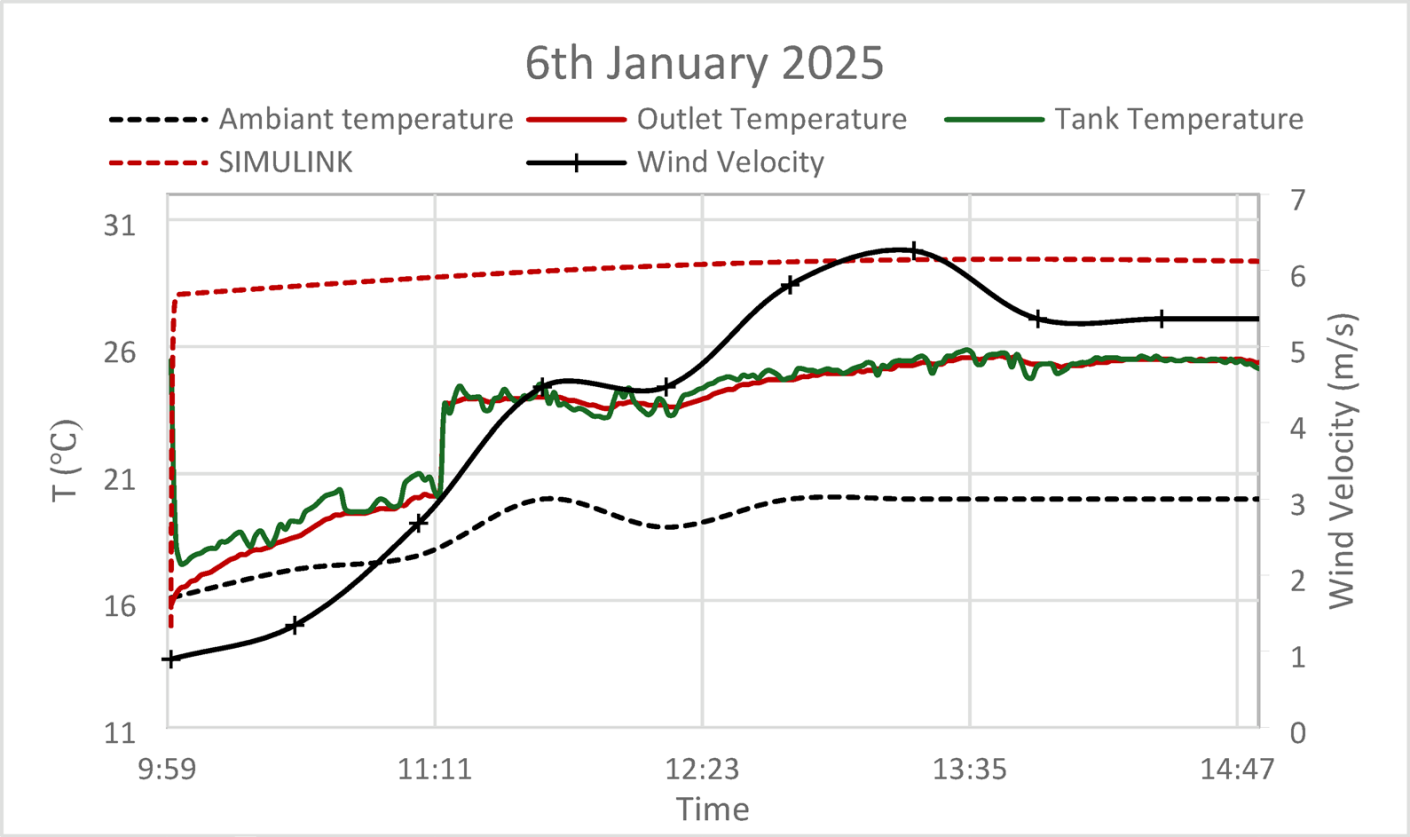
Experimental versus simulink results on 6th January 2025, solar irradiance 930 W/m^2^, wind speed 5 m/s, T_am_ 300 K, and measurement uncertainty: ± 0.5.

Based on the present models both the GA and PSO have nearly similar performance (the results are almost identical). Conversely, for the thermal storage tank, while both GA and PSO deliver similar performance in terms of steady-state error, the PSO controller achieves a faster settling time, making it more suitable for systems requiring quicker thermal adjustments. However, the slightly longer settling time of the GA controller may be more appropriate for larger systems where gradual thermal stabilization is preferred. Regarding the experimental tests the results provide a comparative analysis of ambient temperature, outlet temperature, tank temperature, and wind velocity, alongside predictions from a Simulink model, as shown in Figs. 16, 17 and 18.

During the winter months, the observed lower ambient temperatures (15–18 °C) have a direct impact on system efficiency. While tank and outlet temperatures show increases that parallel with the trend of ambient temperatures, they remain consistently below the values projected by the Simulink model. This phenomenon indicates that a combination of environmental heat losses and diminished solar radiation during winter months significantly constrains the system’s performance. The experimental data reveals that wind velocity fluctuates between 6 and 10 m/s playing a critical role in enhancing convective heat losses from the absorber tube surface by 15–22%, which correlates with the observed temperature drop versus simulations. Consequently, this explains the lower experimental outlet and tank temperatures compared to the estimates provided by the Simulink model, as wind-induced cooling effects are not integrated into standard simulation frameworks.

The analysis points out the relation of high wind speed with decreased temperature differences from both the outlet and tank tests. It may be interpreted that high-wind conditions decrease the thermal efficiency of the system. Moreover, the temperature profile of the tank is stable but lower, showing a limited potential for thermal storage efficiency, probably further reduced by cooling due to wind.

In a nutshell, whereas the Simulink model can be taken as a fundamental theoretical tool in forecasting system behavior, the empirical results underline the critical necessity of taking environmental variables—such as wind speed and seasonal variations—into consideration to increase the precision of modeling and then optimize the whole system design. These results enable the creation of more effective solar heating strategies in practical applications.

## Conclusion and future work

This study has successfully developed, optimized, and validated a novel MPTC for solar water heating applications, demonstrating significant improvements in efficiency and adaptability under varying environmental conditions. By employing an optimized PID controller, the research demonstrated that GA and PSO deliver superior performance in tuning the control parameters of the MPTC. Specifically, GA achieves a lower steady-state error of 0.0000425% versus 0.0014% with PSO and a faster settling time of 13 min versus 14 min for PSO, which is critical for maintaining stable and precise hot-water delivery.

Conversely, the PSO-based controller showed greater effectiveness when applied to the thermal storage tank, offering faster response times but slightly reduced precision. Both methods yield similar control gains with negative K_p_ and K_i_ values, but GA yields a higher K_d_ value, indicating improved responsiveness to dynamic operating conditions. Manual tuning suits rapid prototyping but risks instability.

In conclusion, both the GA and PSO demonstrate distinct advantages in control design optimization. Within this work, GA achieved lower steady-state error and faster settling time, while PSO exhibited smaller overshoot values.

The experimental validation confirms the accuracy of the developed mathematical models and Simulink simulations; however, discrepancies are observed, particularly due to the influence of fluctuating wind velocity, which remains inadequately addressed in existing thermal performance models. These findings emphasize the importance of incorporating robust wind modeling and other real-life environmental factors into future simulations to better reflect practical operating conditions.

Overall, this research underscores the potential of integrating real-time atmospheric data and advanced optimization techniques to enhance the Performance, reliability, and efficiency of solar water heating systems. The insights gained pave the way for further innovation in solar thermal technologies, supporting broader adoption and contributing to sustainable energy solutions. By addressing the identified limitations, this work lays a strong foundation for optimizing the performance and sustainability of next-generation solar water heating systems.

For future work, it is recommended to design an enhanced experimental setup capable of isolating wind effects through improved tank insulation and controlling environmental conditions. Due to funding and time constraints, these refinements could not be achieved in the present study.

## Supplementary Information

Supplementary Information.

## Data Availability

Because the work contains MATLAB routines that the author has created, the datasets created and/or analyzed during the current work are not publicly available, but they are available from the corresponding author upon justifiable request.
